# Endocardial Radiofrequency Ablation vs. Septal Myectomy in Patients With Hypertrophic Obstructive Cardiomyopathy: A Systematic Review and Meta-Analysis

**DOI:** 10.3389/fsurg.2022.859205

**Published:** 2022-04-26

**Authors:** Tao Jiang, Bingyu Huang, Shengqi Huo, Lulu Monica Mageta, Junyi Guo, Jiagao Lv, Li Lin

**Affiliations:** Division of Cardiology, Department of Internal Medicine, Tongji Hospital, Tongji Medical College, Huazhong University of Science and Technology, Wuhan, China

**Keywords:** endocardial radiofrequency ablation, septal myectomy, hypertrophic obstructive cardiomyopathy, systematic review, meta-analysis

## Abstract

**Background:**

Septal myectomy (SM) has been the gold standard therapy for most patients with hypertrophic obstructive cardiomyopathy (HOCM). Endocardial radiofrequency ablation of septal hypertrophy (ERASH) is a novel treatment for septal reduction. We aimed to assess the efficacy and safety between two treatment strategies.

**Methods:**

We searched PubMed, Web of Science, Cochrane Library, and Embase databases to identify relevant studies published up to March 2021. Random-effect models were used to calculate standardized mean difference (SMD) and 95% confidence intervals (CIs) for resting left ventricular outflow tract gradient (LVOTG) and septal thickness.

**Results:**

Twenty-five studies are included in this review, eighteen studies for SM and seven studies for ERASH. During follow-up, there were significant reductions of the mean resting LVOTG in adults (SM groups: SMD = −3.03, 95% CI [−3.62 to −2.44]; ERASH groups: SMD = −1.95, 95% CI [−2.45 to −1.45]) and children (SM groups: SMD = −2.67, 95% CI [−3.21 to −2.12]; ERASH groups: SMD= −2.37, 95% CI [−3.02 to −1.73]) after the septal reduction therapies. For adults, SM groups contributed to more obvious reduction than ERASH groups in interventricular septal thickness (SM groups: SMD = −1.82, 95% CI [−2.29 to −1.34]; ERASH groups: SMD = −0.43, 95% CI [−1.00 to 0.13]). The improvement of the New York Heart Association class was similar in the two groups (SM groups: 46.4%; ERASH groups: 46.7%). The periprocedural mortality in SM and ERASH were 1.1 and 1.8%, respectively.

**Conclusions:**

This systematic review suggests that SM is superior to ERASH in the treatment of HOCM. But for the patients who are at risk for open cardiac surgeries or prefer a less invasive approach, ERASH might be an optional approach.

## Introduction

Hypertrophic cardiomyopathy (HCM) is a genetic disease that occurs in 1 individual out of 500 in the population (1). A significant number of patients with HCM (70%) exhibit left ventricular outflow tract (LVOT) obstruction (2). The pathophysiology of hypertrophic obstructive cardiomyopathy (HOCM) can be explained by the systolic anterior motion of the mitral valve, hypertrophy of the basal septum, mitral–interventricular septal contact, and aberrant papillary muscles (3). Severe LVOT obstruction can result in detrimental symptoms such as dyspnea, chest pain, presyncope, syncope, and predisposing to arrhythmias. The deleterious effects caused by the LVOT obstruction can be eliminated by septal reduction therapies (4).

There is a lack of access to high-quality surgical septal myectomy (SM) because its success relies upon the expertise possessed by the operators, which is unavailable in most hospitals. Recently, there is a novel septal reduction therapy, endocardial radiofrequency ablation of septal hypertrophy (ERASH). It is a minimally invasive method with assuring clinical results up to now (5). There are very few publications currently comparing ERASH and SM. In this systematic review, our aim is to compare efficacy and safety between patients undergoing ERASH and SM.

## Methods

### Search Strategy

Following guidelines of Preferred Reporting Items for Systematic Reviews and Meta-Analyses (PRISMA) (6), we systematically searched PubMed, Web of Science, Cochrane Library, and Embase databases to identify relevant current studies published to March 2021.

#### Search Strategy for Endocardial Radiofrequency Ablation Studies

We used the following mesh terms and free words: “Endocardial radiofrequency ablation OR Percutaneous radiofrequency septal reduction OR Radiofrequency catheter ablation OR Radiofrequency septal reduction OR Radiofrequency ablation, OR Catheter ablation” AND “Cardiomyopathy, Hypertrophic OR Hypertrophic obstructive cardiomyopathy OR Hypertrophic cardiomyopathy, OR Obstructive hypertrophic cardiomyopathy” to identify endocardial radiofrequency ablation studies.

#### Search Strategy for SM Studies

We used the following mesh terms and free words: “Myotomy OR Myectomy, OR Myomectomy” AND “Hypertrophic obstructive cardiomyopathy OR Hypertrophic cardiomyopathy, OR Obstructive hypertrophic cardiomyopathy” to identify SM studies. Since most of ERASH studies were published after January 2016, we limited the publication date of SM studies to 2016 onwards.

### Study Selection

Studies that met the following criteria were included in this review: (1) articles published in English language; (2) studies reporting at least two of the following outcomes: LVOT gradient (LVOTG), septal thickness, or New York Heart Association (NYHA) class after endocardial radiofrequency ablation or SM; (3) SM studies published on or after 1 January 2016. After removing the duplicates, two reviewers (TJ and BH) independently screened the studies for eligibility according to the inclusion criteria. Any disagreements were resolved by discussion with the senior author (LL).

### Data Extraction

We extracted the following data from all included articles: first author, year of publication, country, sample size, mean age, the proportion of males, mean follow-up periods, baseline, and postoperative resting LVOTG, baseline and postoperative septal thickness, baseline and postoperative NYHA class, number of deaths within 30 days, number of deaths after 30 days, causes of deaths, hospital stay, intensive care unit (ICU) stay, permanent pacemaker implantation, and other complications. For studies that only reported median, range, and/or interquartile range for age, resting LVOTG, septal thickness, hospital stay, and ICU stay, we converted these values to mean and SD according to the methods described by Luo et al. (7) and Wan et al. (8).

Two investigators (TJ and BH) individually extracted data from the included articles. Any inconsistencies were resolved by discussion between the two authors. If necessary, we contacted the corresponding authors of eligible studies for more information.

### Quality Assessment

All included studies were independently assessed by two investigators (TJ and BH) for methodological quality using the method published by Murad et al. (9). This assessment covers four perspectives (i.e., selection, ascertainment, causality, and reporting) of methodology. Disagreements in quality appraisal were resolved through consensus.

### Statistical Analysis

Random-effect models were used to calculate standardized mean difference (SMD) and 95% confidence intervals (CIs) for resting LVOTG and septal thickness. Data were analyzed separately for children (<18 years) and adults (≥18 years). Heterogeneity of the studies was evaluated using *I*^2^ statistics (0–100%). Low heterogeneity was defined as *I*^2^ < 50%, moderate heterogeneity was 50% < *I*^2^ < 75%, and *I*^2^ > 75% stood for high heterogeneity. Funnel plot (10) and Egger's regression test (11) were used to determine publication bias. Statistical analyses were performed using Stata version 15.0 (Stata Corporation; College station, Texas, USA).

## Results

### Literature Search

**Figure 1:**
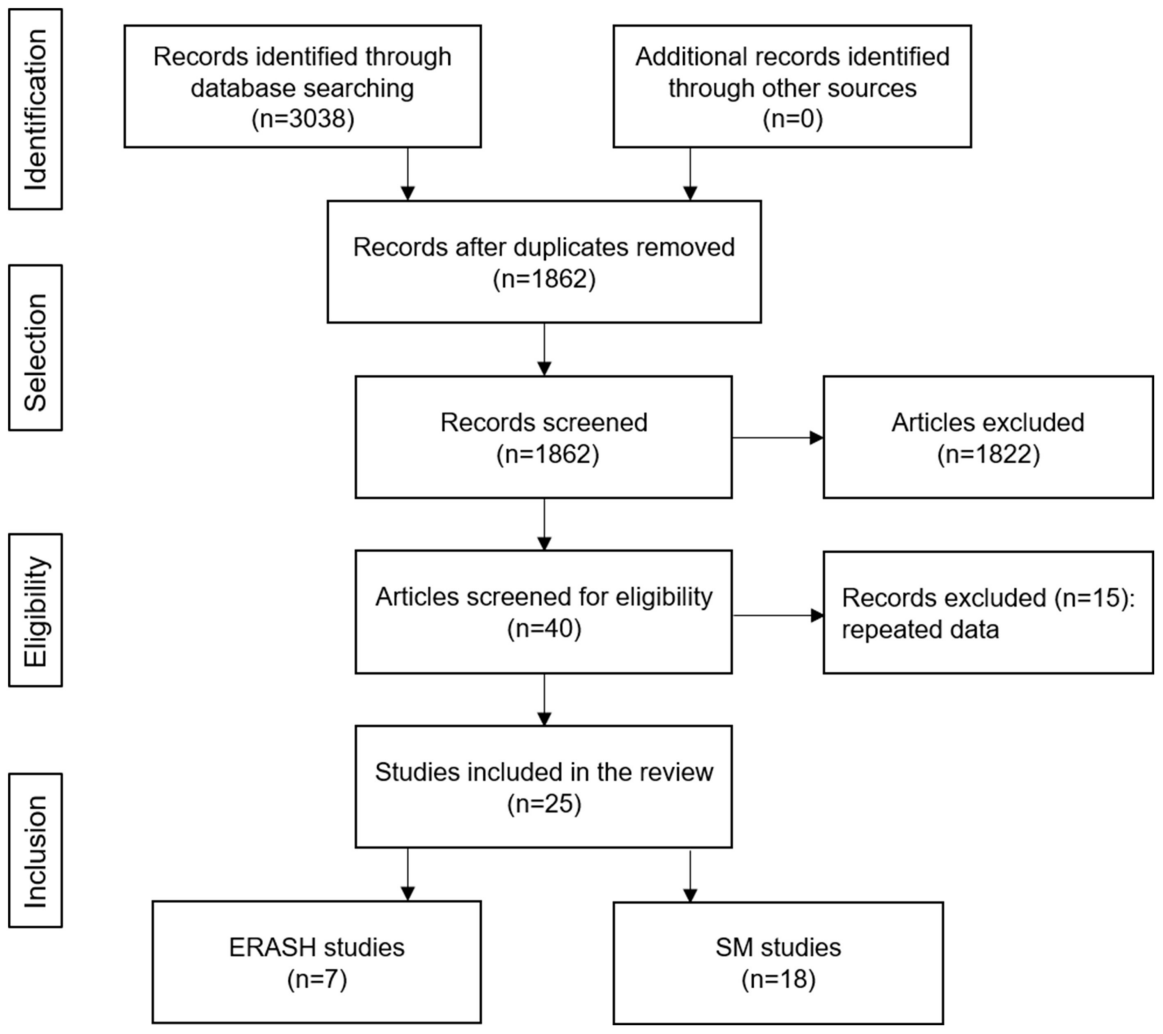
Flowchart of the study selection process. ERASH, endocardial radiofrequency ablation of septal hypertrophy; SM, septal myectomy.

Figure 1 shows the flowchart of the selection procedure for studies. We identified 3,038 articles from PubMed, Web of Science, Cochrane Library, and Embase databases. After removing the duplicates, 1,862 articles remained. And when inclusion and exclusion criteria were applied, 40 studies remained. Further, 15 studies were excluded because of duplicate data with included studies, and 25 studies remained available for the review. All the included studies were observational, seven studies for ERASH and eighteen studies for SM.

### Study Characteristics

Overall, 25 studies were included in this systematic review (Table 1). Eighteen studies were for SM and seven studies were for ERASH. For SM cohorts, five studies (28%) were from European centers (544 patients) (18, 21, 24, 27, 28), five studies (28%) from North American centers (1,187 patients) (12, 15, 16, 20, 25), five studies (28%) from China (650 patients) (13, 14, 17, 26, 29), and the remaining three studies from Argentina (28 patients) (37), Turkey (41 patients) (22), and Bangladesh (21 patients) (23). For ERASH cohorts, three studies (43%) were from European centers (56 patients) (30, 31, 36), two studies (29%) from North American centers (16 patients) (32, 34), and the remaining two studies from China (30 patients) (35) and India (7 patients) (33). Table 2 shows the quality assessment for included studies in four domains with eight explanatory questions.

**Table 1:** Baseline characteristics of the studies included in the review.

**References**	**Country**	**N**	**Age (years)**	**Male (%)**	**Follow up (years)**	**Baseline resting LVOTG (mmHg)**	**Baseline septal thickness (mm)**	**Baseline NYHA class**
**Septal myectomy, age ≥18y**								
Mazine et al. (12)	Canada	25	53.3 ± 10.7	N	1.3	78.4 ± 29.8	21.7 ± 2.5	2.9
Yao et al. (13)	China	139	43.0 ± 15.0	37	5.6	84.0 ± 17.0	22.2	2.7
Lai et al. (14)	China	236	49.3 ± 12.8	57	3.0	79.0 ± 41.7	22.2 ± 7.9	2.4
Rastegar et al. (15)	USA	482	52.0 ± 14.0	57	3.5	56.4 ± 42.4	20.1 ± 4.7	3.0
Vanderlaan et al. (16)	Canada	150	51.6 ± 14.2	62	0.1	67.0 ± 38.0	21.0 ± 4.3	2.8
An et al. (17)	China	118	38.5 ± 14.1	77	5.1	85.8 ± 37.3	33.0 ± 3.7	2.7
Cavigli et al. (18)	Italy	71	48.0 ± 15.0	62	4.2	52.0 ± 31.0	24.0 ± 5.0	2.7
Vrancic et al. (19)	Argentina	28	53.3 ± 13.4	54	1.5	55.1 ± 21.9	21.9 ± 4.5	N
Nguyen et al. (20)	USA	334	64.7 ± 10.0	46	0.6	86.4 ± 25.3	N	3.0
Afanasyev et al. (21)	Russia	345	55.0 ± 13.4	45	3.2	83.4 ± 24.2	24.5 ± 4.6	2.6
Antal et al. (22)	Turkey	41	49.8 ± 13.3	66	3.2	116.7 ± 37.4	23.5 ± 4.1	3.3
Islam et al. (23)	Bangladesh	21	39.8 ± 14.0	67	N	81.6 ± 17.1	20.7 ± 3.9	3.0
Lapenna et al. (24)	Italy	26	59.0 ± 12.0	77	6.0	63.0 ± 20.0	17.0 ± 3.0	2.7
Sun et al. (25)	USA	196	48.4 ± 15.7	50	2.9	49.7 ± 40.3	23.0 ± 7.2	3.1
**Septal myectomy, age < 18y**								
Xu et al. (26)	China	40	11.3 ± 4.3	68	2.0	80.1 ± 33.8	27.2 ± 8.0	2.3
Laredo et al. (27)	France	79	8.3 ± 4.1	72	6.0	104.8 ± 41.6	23.3	2.5
Schleihau et al. (28) cohort 1	Germany	12	0.7 ± 0.3	67	N	94.1 ± 25.7	N	3.0
Schleihau et al. (28) cohort 2	Germany	11	10.8 ± 5.0	55	N	85.4 ± 29.2	N	3.1
Zhu et al. (29)	China	117	11.3 ± 4.7	64	3.2	76.7 ± 28.2	23.7 ± 8.4	2.5
**Endocardial radiofrequency ablation, age ≥18y**								
Lawrenz et al. (30)	Germany	19	60.7 ± 12.0	N	0.5	87.4 ± 34.7	22.6 ± 3.7	3.0
Cooper et al. (31)	UK	5	57.6 ± 9.0	20	0.5	64.3 ± 50.6	18.3 ± 1.9	3.0
Crossen et al. (32)	USA	11	62.0 ± 9.0	36	1.0	66.7 ± 37.9	21.0	3.0
Shelke et al. (33)	India	7	43.7 ± 15.6	71	1.0	81.0 ± 14.8	N	3.0
Beaser et al. (34)	USA	5	61.0	40	0.1	68.7 ± 45.3	19.8 ± 4.5	3.0
Zuo et al. (35)	China	30	45.6 ± 15.7	67	1.0	95.0	23.3 ± 4.1	2.3
**Endocardial radiofrequency ablation, age < 18y**								
Sreeram et al. (36)	UK, Germany	32	10.3 ± 4.3	59	4.0	96.9 ± 27.0	N	N

*LVOTG, left ventricular outflow tract gradient; N, not documented; NYHA, New York Heart Association*.

**Table 2:** Quality assessment of the included studies.

**References**	**Selection**	**Ascertainment**		**Causality**				**Reporting**
	**Does the patients represent the whole experience of the centers**	**Was the exposure adequately ascertained**	**Was the outcome adequately ascertained**	Were other alternative causes that may explain the observation ruled out^*^	Was there a challenge/rechallenge phenomenon^*^	Was there a dose–response effect^*^	**Was follow-up long enough for outcomes to occur**	**Were the cases described with sufficient to allow other investigators to replicate**
**Septal myectomy**								
Mazine et al. (12)	YES	YES	NO	YES	YES	N	YES	YES
Xu et al. (26)	YES	YES	YES	YES	NO	N	YES	YES
Yao et al. (13)	YES	YES	YES	YES	N	N	YES	YES
Lai et al. (14)	YES	YES	YES	YES	N	N	YES	YES
Rastegar et al. (15)	YES	YES	NO	YES	NO	N	YES	YES
Vanderlaan et al. (16)	YES	YES	NO	YES	N	N	NO	YES
An et al. (17)	YES	YES	NO	YES	N	N	YES	YES
Cavigli et al. (18)	YES	YES	YES	YES	YES	N	YES	YES
Laredo et al. (27)	YES	YES	NO	YES	YES	N	YES	YES
Schleihauf et al. (28)	YES	YES	NO	YES	N	N	YES	YES
Vrancic et al. (19)	YES	YES	NO	YES	N	N	YES	YES
Nguyen et al. (20)	NO	YES	NO	YES	N	N	YES	YES
Afanasyev et al. (21)	YES	YES	YES	YES	N	N	YES	YES
Antal et al. (22)	YES	YES	NO	YES	YES	N	YES	YES
Islam et al. (23)	YES	YES	YES	YES	NO	N	N	YES
Lapenna et al. (24)	NO	YES	NO	YES	N	N	YES	YES
Zhu et al. (29)	YES	YES	NO	YES	YES	N	YES	YES
Sun et al. (25)	NO	YES	NO	YES	YES	N	YES	YES
**Endocardial radiofrequency ablation**								
Lawrenz et al. (30)	YES	YES	YES	YES	NO	N	YES	YES
Sreeram et al. (36)	YES	YES	NO	NO	YES	N	YES	YES
Cooper et al. (31)	N	YES	YES	YES	N	N	YES	YES
Crossen et al. (32)	N	YES	YES	YES	YES	N	YES	YES
Shelke et al. (33)	YES	YES	NO	YES	YES	N	YES	YES
Beaser et al. (34)	N	YES	NO	YES	N	N	NO	YES
Zuo et al. (35)	YES	YES	NO	YES	N	N	YES	YES

* *Mostly relevant to cases of adverse drug events*.

*N, not documented*.

### Baseline Characteristics

The baseline characteristics of the studies included in this review are shown in Table 1. The SM cohorts contained a total of 2,471 patients with a mean follow-up of 3.0 years, whereas ERASH comprised a total of 109 patients with a mean follow-up of 1.7 years. The proportions of male patients in SM cohorts and ERASH cohorts were 54.9 and 56.7%, respectively. For adults, the mean age in SM cohorts was 52.3 ± 15.0 years and in ERASH cohorts 52.7 ± 15.4 years. For children, the mean age in SM cohorts was 9.9 ± 5.0 compared with 10.3 ± 4.3 in ERASH. For adults, the mean baseline resting LVOTG for SM was 72.5 ± 37.5 mmHg while for ERASH 77.2 ± 36.2 mmHg. For children, the mean baseline resting LVOTG in SM cohorts and ERASH cohorts were 87.0 ± 35.6 mmHg and 96.9 ± 27.0, respectively. The mean baseline septal thickness was 24.0 ± 6.5 mm for the SM cohorts while 22.6 ± 4.0 mm for ERASH in adults. The mean baseline septal thickness for children was 24.6 ± 8.4 in SM cohorts. For adults, the mean baseline NYHA class for SM cohorts and ERASH cohorts were 2.8 and 3.0, respectively. For children, the mean baseline NYHA class was 2.5 in SM cohorts.

### Clinical Outcomes

The clinical outcomes after operation are summarized in Table 3.

**Table 3:** Clinical outcomes.

**Author**	**Post-procedural resting LVOTG (mmHg)**	**Post-procedural septal thickness (mm)**	**Post-procedural NYHA class**	**Number of deaths <30 days**	**Number of deaths >30 days**	**Causes of Deaths**	**PPMI (%)**	**Complications**	**Hospital Stay (d)**	**ICU stay (d)**
**Septal myectomy, age ≥18 y**										
Mazine et al. (12)	16.5 ± 10.5	14.7 ± 2.1	N	0	1	Non-cardiac	20.0	AF, AVB, LBBB, RI	9.6 ± 9.2	N
Yao et al. (13)	6.0 ± 3.0	16.8	1.7	0	3	Cardiac, Unknown	5.8	AF, AVB, LBBB	10.5 ± 5.0	3.0 ± 3.0
Lai et al. (14)	11.8 ± 10.0	16.5 ± 3.8	1.6	4	1	Sepsis, HF, LCOS, Stroke	2.5	LBBB	N	2.0 ± 2.5
Rastegar et al. (15)	1.2 ± 7.0	N	1.4	4	11	Cardiogenic shock, PT, Respiratory failure, HF, Systolic dysfunction, Non-cardiac	8.9	AF, VSD	6.2 ± 3.1	N
Vanderlaan et al. (16)	11.0 ± 7.0	10.4 ± 2.6	N	1	0	HF	5.3	N	6.0 ± 1.5	1.4 ± 0.7
An et al. (17)	11.8 ± 10.1	17.0 ± 7.8	N	0	5	SCD, HF	N	N	N	N
Cavigli et al. (18)	11.0 ± 10.0	20.0 ± 5.0	1.6	1	3	HF, Non-cardiac	8.5	N	N	N
Vrancic et al. (19)	8.3 ± 5.4	13.2 ± 3.0	N	0	1	Stroke	14.3	AF	5.4 ± 2.7	N
Nguyen et al. (20)	0.0 ± 0.0	N	1.6	0	N	None	3.9	N	6.0 ± 1.5	N
Afanasyev et al. (21)	16.2 ± 8.5	19.3 ± 4.3	1.3	6	10	Stroke, MI, MOF, PT, PE, SCD, Thromboembolism, Non-cardiac	8.4	AVB, PE, PT, RI, VSD	N	N
Antal et al. (22)	22.5 ± 16.3	17.4 ± 3.1	N	1	0	LCOS	2.4	AF, VSD	N	N
Islam et al. (23)	8.9 ± 2.5	15.0 ± 1.9	1.0	0	0	None	4.8	N	N	N
Lapenna et al. (24)	9.4 ± 3.9	N	1.7	1	2	LCOS, HF, SCD	3.8	N	N	0.7 ± 0.2
Zhu et al. (29)	14.4 ± 12.1	17.9 ± 7.9	N	1	0	HF, MOF	2.6	AVB, RBBB, LBBB	N	N
Sun et al. (25)	9.1 ± 14.2	15.7 ± 4.5	N	2	6	MOF, SCD	1.5	N	N	N
**Septal myectomy, age < 18 y**										
Xu et al. (26)	14.7 ± 11.5	16.7 ± 6.0	1.2	0	1	SCD	2.5	N	N	1.5 ± 1.0
Laredo et al. (27)	11.0 ± 6.6	N	1.3	5	3	HF, MI	N	AVB, AVI, VSD	15.2 ± 18.6	8.1 ± 17.4
Schleihau et al. (28) cohort 1	53.3 ± 38.5	N	1.9	1	1	MOF, SCD	0	N	24.0 ± 19.0	N
Schleihau et al. (28) cohort 2	8.7 ± 11.6	N	2.1	0	1	HF	9.1	AVB	17.0 ± 6.0	N
Zhu et al. (29)	14.4 ± 12.1	17.9 ± 7.9	N	1	0	HF, MOF	2.6	AVB, RBBB, LBBB	10.7 ± 14.8	2.3 ± 2.5
**Endocardial radiofrequency ablation, age ≥18 y**										
Lawrenz et al. (30)	26.5 ± 22.0	21.4 ± 3.4	1.6	0	0	None	21.1	PT	N	N
Cooper et al. (31)	12.3 ± 2.5	16.8 ± 2.5	1.8	1	0	Retroperitoneal hemorrhage	N	LBBB, PE	N	N
Crossen et al. (31)	10.0 ± 5.4	20.0	1.8	0	0	None	18.2	AVB	1.3 ± 0.6	N
Shelke et al. (33)	42.9 ± 24.2	N	1.6	0	0	None	0	PE	N	N
Beaser et al. (34)	8.5 ± 15.1	N	1.4	0	0	None	N	N	N	N
Zuo et al. (35)	12.5	14.4 ± 2.3	N	0	0	None	0	PT	N	N
**Endocardial radiofrequency ablation, age < 18 y**										
Sreeram et al. (36)	32.7 ± 27.1	N	N	1	1	Acute left ventricular dysfunction; Arrhythmia	6.3	AVB, VF	1-2 d	N

*AF, atrial fibrillation; AVB, atrioventricular block; AVI, aortic valve injury; HF, heart failure; ICU, Intensive care unit; LBBB, left bundle branch block; LCOS, low cardiac output syndrome; LVOTG, left ventricular outflow tract gradient; MI, myocardial infarction; MOF, multi-organ failure; N, not documented; NYHA, New York Heart Association; PE, pulmonary edema; PPMI, permanent pacemaker implantation; PT, pericardial tamponade; RBBB, right bundle branch block; RI, renal insufficiency; SCD, sudden cardiac death; VF, ventricular fibrillation; VSD, ventricular septal defect*.

#### Primary Outcomes in Adults (Age ≥ 18 Years)

**Figure 2:**
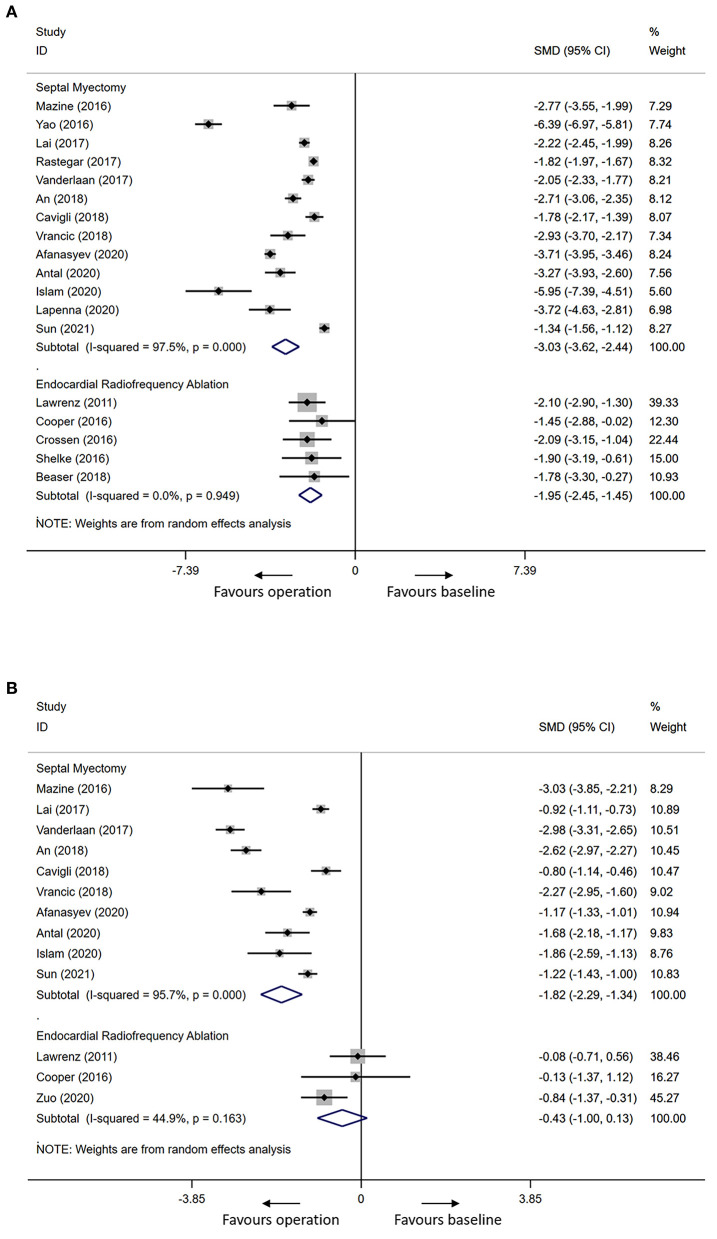
Forest plots for the primary outcomes in adults after septal myectomy or endocardial radiofrequency ablation. **(A)** Forest plot for the resting LVOTG. **(B)** Forest plot for the interventricular septal thickness. LVOTG, left ventricular outflow tract gradient.

The mean resting LVOTG reduced from 72.5 ± 37.5 to 7.9 ± 10.4 mmHg in SM cohorts (mean follow-up period 2.9 years) and from 77.2 ± 36.2 to 21.7 ± 20.8 mmHg in ERASH (mean follow-up period 0.6 years) after the procedure. Figure 2A shows the meta-analysis for the change of resting LVOTG after the procedure. Two groups both had significant efficacy in the change of resting LVOTG (SM groups: SMD = −3.03, 95% CI [−3.62 to −2.44]; ERASH groups: SMD = −1.95, 95% CI [−2.45 to −1.45]). ERASH groups (mean follow-up period 0.8 years) reported a smaller reduction than SM groups in interventricular septal thickness by 5.5 mm (24.3%) in contrast to a 7.4-mm (30.0%) reduction in SM (mean follow-up period 2.9 years). Figure 2B shows the meta-analysis for the change of interventricular septal thickness after the procedure. But only the differences in SM groups were statistically significant (SM groups: SMD = −1.82, 95% CI [−2.29 to −1.34]; ERASH groups: SMD = −0.43, 95% CI [−1.00 to 0.13]). During follow-up, most of the patients in both groups were in NYHA class I or II. The mean NYHA class in SM groups (mean follow-up period 3.0 years) was 1.5 and in ERASH groups (mean follow-up period 0.6 years) was 1.6. The improvement of the NYHA class was similar in the two groups (SM groups: 46.4%; ERASH groups: 46.7%).

#### Primary Outcomes in Children (Age <18 Years)

**Figure 3:**
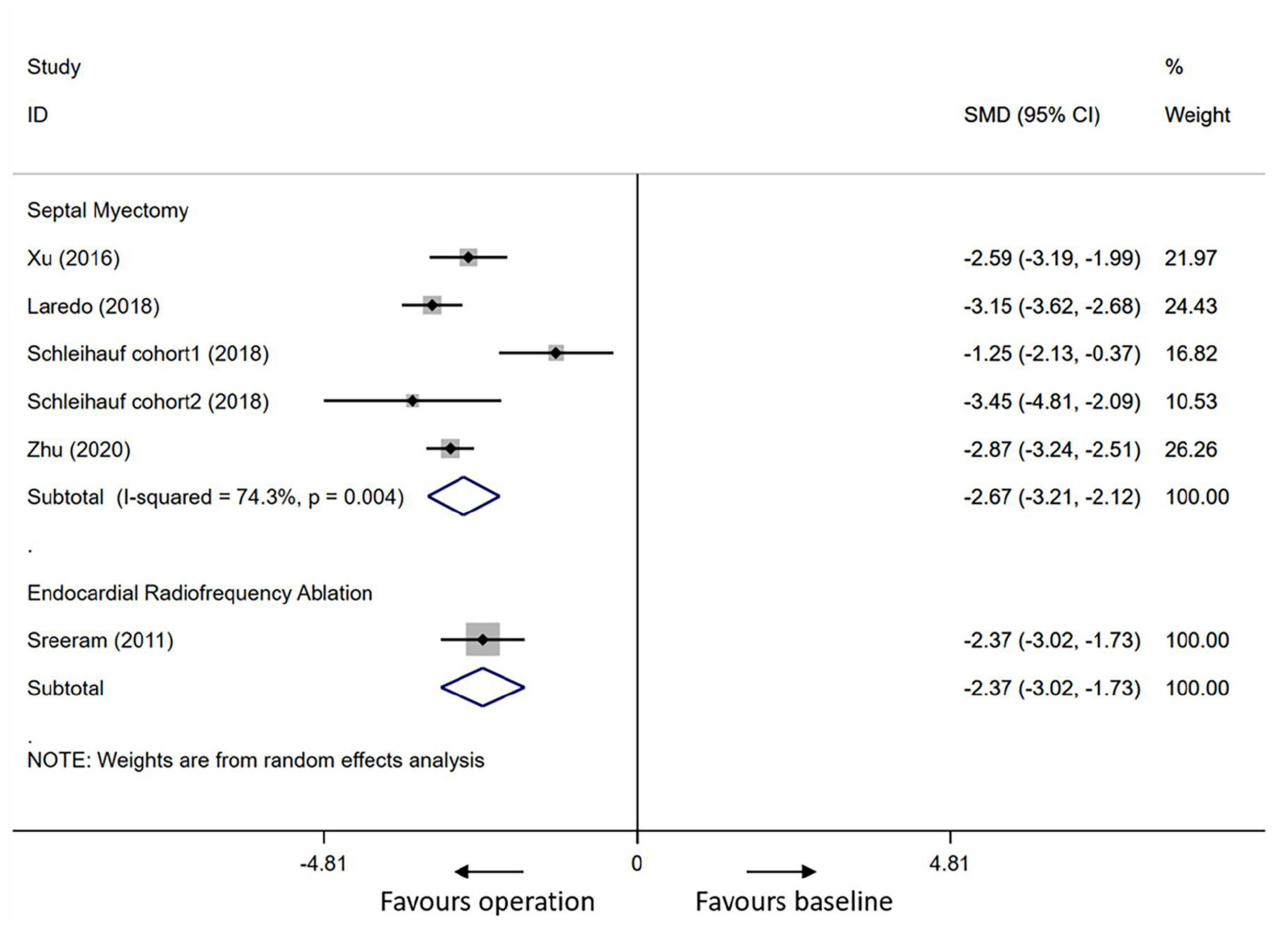
Forest plot for the resting LVOTG in children after septal myectomy or endocardial radiofrequency ablation. LVOTG, left ventricular outflow tract gradient.

The mean resting peak LVOTG reduced from 87.0 ± 35.6 to 15.0 ± 15.6 mmHg in SM cohorts (mean follow-up period 3.9 years) and from 96.9 ± 27.0 to 32.7 ± 27.1 mmHg in ERASH (mean follow-up period 4.0 years) after the procedure. As shown in Figure 3, the differences in SM and ERASH groups were both statistically significant (SM groups: SMD = −2.67, 95% CI [−3.21 to −2.12]; ERASH groups: SMD = −2.37, 95% CI [−3.02 to −1.73]). SM groups reported the reduction of interventricular septal thickness was 7.0 mm (mean follow-up period 2.9 years). The mean improvement of NYHA class in SM groups was 1.1 (mean follow-up period 4.7 years). No studies reported interventricular septal thickness and NYHA class in ERASH groups.

#### Secondary Outcomes

Postoperative hospital stay time and ICU stay in SM groups were 7.7 ± 7.7 days and 2.0 ± 2.3 days, respectively. But in ERASH groups, postoperative hospital stay time was only 1–2 days (32, 36). The 30-day mortality in SM and ERASH groups were 1.1 and 1.8%, respectively. Permanent pacemaker implantation was necessary for 5.9% of patients after SM and 7.3% after ERASH. Perioperative arrhythmia events included atrial fibrillation (AF) and ventricular fibrillation (VF). One study (14%) from the ERASH cohorts documented the incidence of VF, and five studies (28%) from the SM cohorts documented postoperative AF and VF. Left bundle branch block (LBBB) was mainly documented in four studies (22%) of SM and one study (14%) in ERASH. Two studies (29%) documented atrioventricular block (AVB) in ERASH groups and six studies (33%) documented AVB in SM cohorts. Two studies (11%) documented right bundle branch block (RBBB) in SM cohorts. The ERASH groups reported other perioperative complications such as pulmonary edema and pericardial tamponade. Complications recorded by SM cohorts mostly were ventricular septal defect (VSD) and renal insufficiency reported by six studies (33%) and two studies (11%), respectively. Pericardial tamponade and pulmonary edema were reported by two studies (11%) and one study (5%), respectively. Aortic valve injury was reported by only one study (6%).

The mortality after 30 days in SM groups and ERASH groups were 2.7 and 0.9%, respectively, during the follow-up periods. Reoperation was more frequent in ERASH cohorts involving 11.6% of patients, whereas for SM was 1.0%.

### Heterogeneity

In adults, the heterogeneity for mean resting LVOTG and interventricular septal thickness in SM groups was high (*I*^2^ = 97.5%, *p* = 0.000 for mean resting LVOTG; *I*^2^ = 95.7%, *p* = 0.000 for interventricular septal thickness); the heterogeneity test revealed that there was low heterogeneity for mean resting LVOTG and interventricular septal thickness in ERASH groups (*I*^2^ = 0.0%, *p* = 0.949 for mean resting LVOTG; *I*^2^ = 44.9%, *p* = 0.163 for interventricular septal thickness). In children, heterogeneity of the analysis for mean resting LVOTG in SM groups was moderate (*I*^2^ = 74.3%, *p* = 0.004).

### Publication Bias

**Figure 4:**
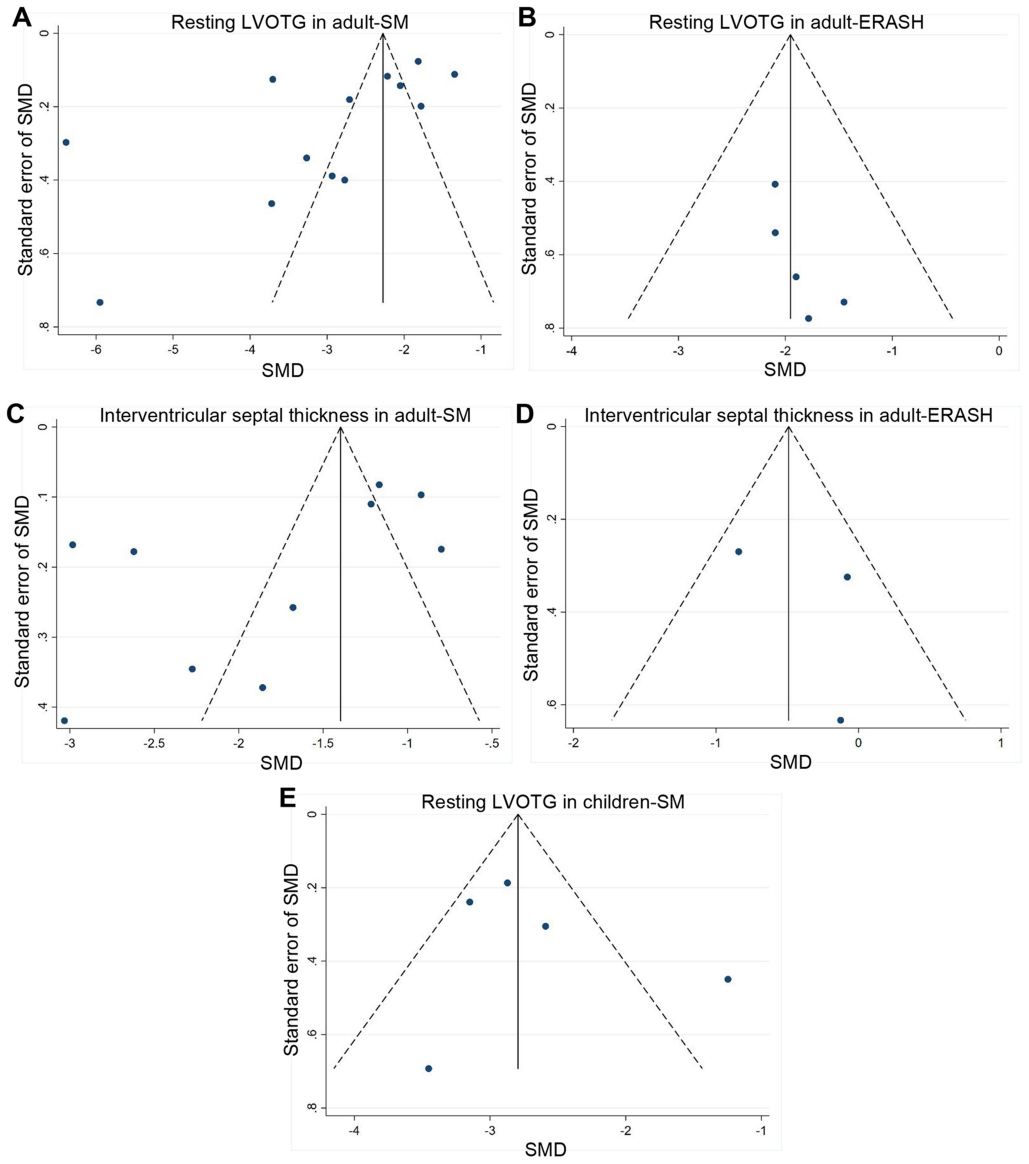
Funnel plots for publication bias. **(A,B)** Funnel plots for the resting LVOTG in adult-SM groups **(A)** and adult-ERASH groups **(B)**. **(C,D)** Funnel plots for the interventricular septal thickness in adult-SM groups **(C)** and adult-ERASH groups **(D)**. **(E)** Funnel plot for the resting LVOTG in children-SM groups. ERASH, endocardial radiofrequency ablation of septal hypertrophy; LVOTG, left ventricular outflow tract gradient; SM, septal myectomy; SMD, standardized mean difference.

We evaluated publication bias by the Funnel plot and Egger's test. Funnel plots for the resting LVOTG in adult-SM groups (Figure 4A) and adult-ERASH groups (Figure 4B) were relative symmetry, and publication bias was not found by the Egger's test (*p* = 0.052 for SM; *p* = 0.095 for ERASH). Funnel plots for interventricular septal thickness in adult-SM groups (Figure 4C) and adult-ERASH groups (Figure 4D) were also symmetry, and publication bias was not found by the Egger's test (*p* = 0.081 for SM; *p* = 0.636 for ERASH). The publication bias for the resting LVOTG in children-SM groups was not obvious (Figure 4E; Egger's test *p* = 0.546).

## Discussion

For patients whose symptoms cannot be relieved by optimal medications, septal reduction is necessary. In experienced centers, ERASH and SM result in a significant reduction of the LVOTG as well as the amelioration of clinical symptoms in patients.

### Geographical Location

Based on the currently available publications, it seems that ERASH is mainly being carried out in Germany and the UK (30, 31, 36), and SM is now performed more frequently by China centers (13, 14, 17, 26, 29). The surgical SM centers in Europe are not as active as they used to be. Based on this fact, the postoperative outcomes might not be well-represented primarily from the small volume centers. The European centers are instead performing alcohol septal ablation (ASA) in large number populations (38).

### Expertise

Septal myectomy requires unique skills that are not available in most of the centers. To acquire such surgical expertise, larger patient volume is needed to be executed (39). For over two decades, radiofrequency ablation has been used for the treatment of arrhythmias (40); hence, the tools and expertise are widely available. Also, the introduction of ASA in 1995 as a septal reduction therapy makes non-surgical septal reduction an attractive alternative for HOCM (41).

### Hospital Length of Stay

Endocardial radiofrequency ablation of septal hypertrophy is associated with the shorter hospital stay (<2 days) than SM (7.7 ± 7.7 days), and the possible causes are the healing of the chest wound as well as the intrathoracic cannula. In addition, the patients undergoing SM needed to stay in the ICU (2.0 ± 2.3 days) due to SM involving sternotomy and the use of cardiopulmonary bypass (13, 14, 16, 24, 26, 27). A shorter hospital stay for ERASH may reduce the hospital costs. Perioperative care is critical for postoperative rehabilitation of patients (42). Compared to ERASH, the minimally invasive operation, SM requires more specialized postoperative care, which is easier to implement in large hospitals.

### Clinical Outcomes

Although there was minimal septal thickness change in the ERASH groups vs. SM groups (24.3 vs. 30.0%) in adults, it also resulted in a compelling reduction of the LVOTG (71.9%). LVOTG relief is more reliable and complete in patients undergoing SM (89.2%). NYHA class improved from 3.0 at baseline to 1.6 during follow-up period in ERASH groups, and from 2.8 to 1.5 in SM groups. The improvement of NYHA class was similar in the ERASH groups and SM groups (46.7 vs. 46.4%). Notably, the follow-up period was significantly longer in SM groups compared to ERASH groups. Lawrenz et al. (30) observed a sustained reduction of LVOTG and improvement of 6-min walking distance over time within 6 months after ERASH. The study by Crossen et al. (32) also showed that postoperative LVOTG gradually decreased within a year after ERASH. Beneficial effects of ERASH procedure may be more pronounced with longer follow-up period. However, longer follow-up period may also increase the probability of recurrence. Thus, the long-term effects of ERASH require further research.

New pieces of evidence have shown that SM is associated with good outcomes in specialized centers. They incur <1% of complications such as early deaths, cerebrovascular accidents, ventricular arrhythmias, valve injury, and VSDs (43). Early mortality after SM procedure was higher for the patients in small centers (1–3%) than the patients in specialized centers (<1%) (14, 18, 21, 22, 24). There was no perioperative death observed in five out of seven ERASH studies (30, 32–35), which illustrated the safety of ERASH. Two studies reported that two patients appeared acute pulmonary edema immediately following ablation, which may be related to fluid overload due to repeated irrigation during the procedure (31, 33). The edema can be avoided by the use of diuretics, fewer volumes of fluids for irrigation of the catheter tip, and meticulous use of anticoagulation. One patient developed severe retroperitoneal hemorrhage after procedure and ultimately died due to mesenteric ischemia (31). Modi et al. (44) demonstrated that post-procedural mortality ranged from 0.4 to 3.0%, which were some associated with retroperitoneal hemorrhage in the course of radiofrequency ablation for ischemic ventricular tachycardia. Based on the likeness of the ablation techniques, this could be the nearest representation of procedural risks in ERASH.

Septal reduction is more complicated in children compared to the adult populations. Due to the small nature of the aortic annulus, the surgical operation can lead to valve injury or incomplete myectomy (45). Current studies showed that SM achieved better LVOTG relief but resulted in more perioperative deaths and complications (27, 28, 36). So ERASH may be a better alternative for children.

Septal myectomy and other concomitant procedures can be performed simultaneously with a low likelihood of risks and satisfactory results. Such procedures can be used in the management of primary mitral valve disease, papillary muscle abnormalities, aortic valve disease, and atrial arrhythmias (43). ERASH does not allow for additional procedures to be performed.

Septal myectomy requires open cardiac surgery, and the risk factors include advanced age, the presence of multiple comorbidities (e.g., chronic obstructive pulmonary disease, diabetes mellitus, and hypertension). ERASH may be considered in patients who are at risk for open cardiac surgeries and those with advanced age.

An essential part of the treatment of hypertrophy cardiomyopathy is to estimate the risk of sudden death and the indication of implantable cardioverter defibrillator (ICD) implantation according to the current guidelines (46). There are no ERASH studies currently determining the rate of occurrence and survival of sudden cardiac death (SCD). Vriesendorp et al. (47) demonstrated the risk of SCD is lower in SM as compared to medical therapy. Rigopoulos et al. (46) suggested that the risk of SCD could be reduced after septal reduction therapies like SM or ASA. LVOT obstruction is speculated to be one of the risk factors for SCD.

A major drawback of ERASH is the requirement of pacemaker implantation, and ERASH is more associated with complete conduction blocks compared with SM (9.5 vs. 5.9%). But with the application of three-dimensional mapping system and intracardiac echocardiography, conduction blocks can be largely attenuated by allowing accurate localization of mitral–interventricular septal area (31). If larger cohorts can be performed, ERASH can be developed just like ASA, which has been widely used in recent years. The shorter recovery, availability of expertise, and its minimally invasive nature makes it an alternative treatment.

### Heterogeneity

The result of heterogeneity test showed that there was significant heterogeneity in SM groups (*I*^2^ > 50%, *p* < 0.05). We did not detect publication bias in SM groups (Egger's test *p* > 0.05), suggesting that the heterogeneity might not be due to publication bias. The sources of heterogeneity were diverse, such as patient characteristics (ethnicity, gender, age, etc.), surgical techniques, methodological, and statistical heterogeneity, which may lead to the meta-analysis heterogeneity. Although the heterogeneity in ERASH groups was low (*I*^2^ < 50%, *p* > 0.05), relatively small number of included studies might be one explanation for the low heterogeneity.

### Limitations

Our review has several limitations. First, there is conspicuous heterogeneity between comparison groups of our review; hence, results should be interpreted with caution. Second, all studies included in the review were observational. Last, the study population sizes were small for ERASH studies, and the follow-up durations were relatively short compared to SM cohorts, which may be prone to bias.

## Conclusions

Indeed, our review shows that SM is associated with better clinical outcomes than ERASH. However, for patients who are at risk for open cardiac surgeries or prefer a less invasive approach, ERASH might be an optional approach. Trials with larger sample sizes and longer follow-up periods are needed for ERASH to characterize its efficacy and safety.

## Data Availability Statement

The raw data supporting the conclusions of this article will be made available by the authors, without undue reservation.

## Author Contributions

LL, JL, and TJ designed the work. TJ performed study selection, data collection, data extraction, quality assessment, data analysis, and writing the manuscript. BH helped study selection, data collection, data extraction, and quality assessment. SH, LM, and JG contributed to the interpretation of data. LL and JL reviewed and edited the paper. All authors read and approved the final manuscript.

## Funding

This work was supported by the Science and Technology Project Foundation of Wuhan (grant number 2019020701011439), and the National Natural Science Foundation of China (Grant No. 82070396).

## Conflict of Interest

The authors declare that the research was conducted in the absence of any commercial or financial relationships that could be construed as a potential conflict of interest.

## Publisher's Note

All claims expressed in this article are solely those of the authors and do not necessarily represent those of their affiliated organizations, or those of the publisher, the editors and the reviewers. Any product that may be evaluated in this article, or claim that may be made by its manufacturer, is not guaranteed or endorsed by the publisher.
